# Molecular and physiological responses to salt stress in salinity-sensitive and tolerant *Hibiscus rosa-sinensis* cultivars

**DOI:** 10.1186/s43897-023-00075-y

**Published:** 2023-12-19

**Authors:** Alice Trivellini, Giulia Carmassi, Guido Scatena, Paolo Vernieri, Antonio Ferrante

**Affiliations:** 1 Department of Agriculture, Food and Environment, University of Pisa, Pisa, Italy; 2 Italian Institute for Environmental Protection and Research – ISPRA, Via del Cedro 38, 57122 Leghorn, Italy; 3 Department of Agricultural and Environmental Sciences, Università Degli Studi Di Milano, Via Celoria 2, 20133 Milan, Italy

**Keywords:** Flower, ABA, Ion leakage, Petals, Proline, Transcriptome

## Abstract

Ornamental plants are used to decorate urban and peri-urban areas, and during their cultivation or utilisation, they can be exposed to abiotic stress. Salinity is an abiotic stress factor that limits plant growth and reduces the ornamental value of sensitive species. In this study, transcriptomic analysis was conducted to identify genes associated with tolerance or sensitivity to salinity in two hibiscus (*Hibiscus rosa-sinensis* L.) cultivars, ‘Porto’ and ‘Sunny wind’. The physiological and biochemical parameters of plants exposed to 50, 100, or 200 mM NaCl and water (control) were monitored. Salinity treatments were applied for six weeks. After four weeks, differences between cultivars were clearly evident and ‘Porto’ was more tolerant than ‘Sunny wind’. The tolerant cultivar showed lower electrolyte leakage and ABA concentrations, and higher proline content in the leaves. Accumulation of Na in different organs was lower in the flower organs of ‘Porto’. At the molecular level, several differential expressed genes were observed between the cultivars and flower organs. Among the highly expressed DEGs, coat protein, alcohol dehydrogenase, and AP2/EREBP transcription factor ERF-1. Among the downregulated genes, GH3 and NCED were the most interesting. The differential expression of these genes may explain the salt stress tolerance of ‘Porto’.

**Graphical Abstract:**

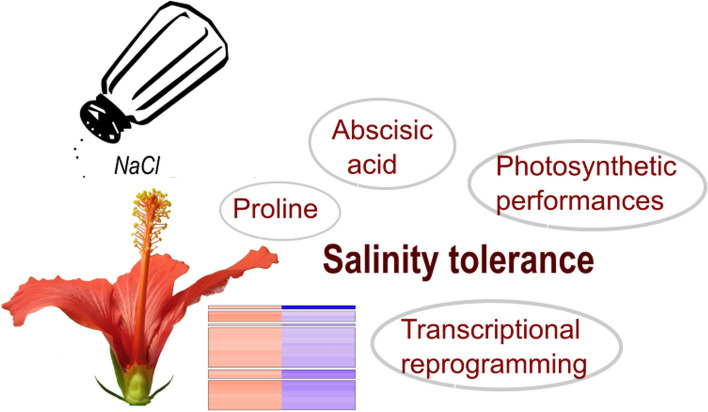

**Supplementary Information:**

The online version contains supplementary material available at 10.1186/s43897-023-00075-y.

## Core

In this study a comparative physiological, biochemical, and transcriptomic analyses was performed to identify conserved or divergent gene expression patterns associated with salt tolerance between two hibiscus cultivars. Comparing of transcriptomes of salt-tolerant ‘Porto’ and –sensitive ‘Sunny wind’ cultivars indicated the different transcriptome might be important reasons of the tolerance.

## Gene & accession number

Transcriptome data generated from this study have been deposited in Genebank database under accession No. [PRJNA325155]. A list of genes used in the qRT-PCR analysis can be found in Supplementary Table S4.

## Introduction

Abiotic stress can dramatically reduce the growth and development of horticultural crops (Mariani and Ferrante 2017; Ferrante and Mariani 2018). Salinity induces a decline in biomass, yield, and quality loss depending on its duration (Toscano et al. 2019). In the ornamental sector, the quality of potted flowering plants is defined by number of open flowers and buds present, which in turn depends on flower longevity and turnover (Ferrante et al. 2015). Ornamental plants can be exposed to salinity stress during cultivation or after production in the utilisation environment, such as along the coast close to the seaside or in a geographic area with high salinity in soil or irrigation water (Yeo 1998). The salinity tolerance threshold differs among ornamental species (Toscano et al. 2020). It is well known that glycophytes are sensitive to salinity and halophyte are tolerant. However, plants can have different degrees of tolerance depending on the strategies they use to counteract salinity stress. Salinity stress can range from 50 to 250 mM NaCl. Most flower species are sensitive to high salt concentration. If exposed to excessive salinity, these species may experience reduced flower growth, diminished flowering, or even death. Salt-sensitive flowers typically struggle to take up water from saline soils, leading to dehydration and reduced growth (Cabot et al. 2014).

Photosynthesis, together with cell growth, is among the primary processes affected by salinity stress and can be indirectly monitored using chlorophyll a fluorescence determination. This non-destructive tool has been used to evaluate the tolerance of ornamental plants to saline aerosols in several species (Ferrante et al. 2011). Plants under salinity reduce the light use efficiency, and chlorophyll parameters such as active reaction centres, electron flux density, performance index, and maximum quantum efficiency of photosystem II decline, while the dissipation of energy as heat increases. These parameters can be used to evaluate salinity tolerance in ornamental species (Ferrante et al. 2011).

Salinity stress in ornamental plants can lead to various biochemical changes, as they adapt to and respond to high salt levels in their environment. The most important biochemical changes observed in ornamentals under salinity stress include ion imbalance, accumulation of reactive oxygen species (ROS), osmotic adjustment, activation of detoxification enzymes, and photosynthesis reduction. Ion imbalance is a common effect of salinity stress that disrupts ion balance in plants, particularly by increasing the concentration of sodium ions (Na^+^) in the soil and subsequently in plant tissues. This enhanced sodium level can interfere with the uptake and assimilation of essential nutrients such as potassium (K^+^), calcium (Ca^2+^), and magnesium (Mg^2+^), leading to nutrient imbalances (Mittler 2002; García-Caparrós and Lao 2018).

ROS, such as superoxide radicals (O_2_^−^) and hydrogen peroxide (H_2_O_2_), are highly reactive molecules that can cause oxidative damage to cellular components including lipids, proteins, and nucleic acids (Van Breusegem and Mittler 2023). ROS reduction and cell protection can be achieved by ROS scavenging systems. This system consists of numerous enzymatic antioxidants such as superoxide dismutase (SOD), catalase (CAT), peroxidase (POD), and ascorbate peroxidase (APX), which scavenge ROS and minimise oxidative damage (Flowers and Colmer 2008).

At the cellular level, salinity can be mitigated by osmotic adjustment. Osmolytes accumulation prevents cellular dehydration and subside osmotic stress caused by salinity by lowering cellular water potential and thereby maintaining a favorable gradient for water uptake from soil to root (Liang et al. 2018). Plants synthesize proline, soluble sugars, glycine, betaine, and other osmolytes to promote osmotic balance, maintain cell turgor pressure and cellular hydration at the cellular level (Liang et al. 2018).

Salinity affects physiological dynamics of photo- synthesis. Salinity stress often impairs photosynthetic activity in ornamental plants. This can lead to a reduction in chlorophyll content, stomatal closure, and decreased photosynthetic rates, which negatively impacts plant growth and development (Hasanuzzaman et al. 2018; Parida and Das 2005). Salinity stress influences the balance of various plant hormones including abscisic acid (ABA), ethylene, and jasmonic acid (Chen and Pang 2023). These hormones play critical roles in regulating plant responses to stress, including ion transport, stomatal closure, osmotic adjustment, and growth inhibition (Mahajan and Tuteja 2005). Increased ethylene production is strongly associated with increased salinity tolerance. However, it must be sure that there are no other sources of stress, since ethylene enhancement is a generic response to biotic and abiotic stresses in plants (Ferrante 2023).

Salinity stress in ornamental plants can induce various molecular changes that affect gene expression, protein synthesis, and signalling pathways. Molecular changes observed in ornamental plants under salinity stress may be associated with tolerance or sensitivity. Salinity stress causes major alterations in the expression patterns of genes involved in diverse physiological and regulatory pathways in many ornamental plants (Toscano et al. 2023). Certain genes involved in ion transport, osmotic regulation, stress signalling, and antioxidant defence mechanisms are upregulated or downregulated in response to salinity stress (Shavrukov 2013). Salinity stress can also induce epigenetic changes, such as DNA methylation and histone modifications, which can alter gene expression patterns and affect stress tolerance in plants. These modifications can provide long-term memory of stress exposure and influence the subsequent responses to salinity stress (Chinnusamy et al. 2007).

Salinity stress triggers phosphorylation and other post-translational modifications of proteins involved in stress signalling, ion transport, and osmotic regulation. These modifications can regulate protein activity, localisation, and interactions, thereby influencing the response of plants to salinity stress (Damaris and Yang 2021).

These molecular changes highlight the complex and dynamic responses of ornamental plants to salinity stress at the molecular level, allowing them to adapt and survive in challenging environments (Trivellini et al. 2016a, b; Toscano et al. 2023).

The aim of this study was to unravel the molecular mechanisms underlying the salt stress response in hibiscus plants via integrated comparative physiological, biochemical, and transcriptomic analyses to identify conserved or divergent gene expression patterns associated with salt tolerance between the two cultivars. In detail, two *Hibiscus rosa-sinensis* L. cultivars were compared in terms of their growth and physiological responses to salt stress. Microarray analysis using a custom microarray (Trivellini et al. 2016a, b) was then performed to assess their global gene regulation by salt stress in flower tissues, which will serve as the first step towards understanding the mechanism underlying transcriptional reprogramming in flowers under salinity conditions in this ornamental species. The results should provide a comprehensive view of the genes and pathways involved in salinity responses and help identify potential target genes or mechanisms for improving salt tolerance in ornamental crops and other horticultural species.

## Results

The effect of salinity was studied considering biomass accumulation, physiological, biochemical, and molecular changes in two cultivars and considering different flower organs. The effects of salinity on visual appearance and ornamental quality were represented by a reduction in flower weight and number. Turnover of flower production was also impaired. Salinity affects reproductive organ formation The ‘Sunny wind’ after four weeks under 200 mM NaCl saline irrigation stopped flower opening whereas “Porto” continued to produce and showing open flowers. In ‘Sunny wind’ the persistence of stress led to plant death after six weeks under 200 mM NaCl treatment and 25% of plants died.

### Physiological and biochemical determinations

#### Biomass accumulation

**Fig. 1:**
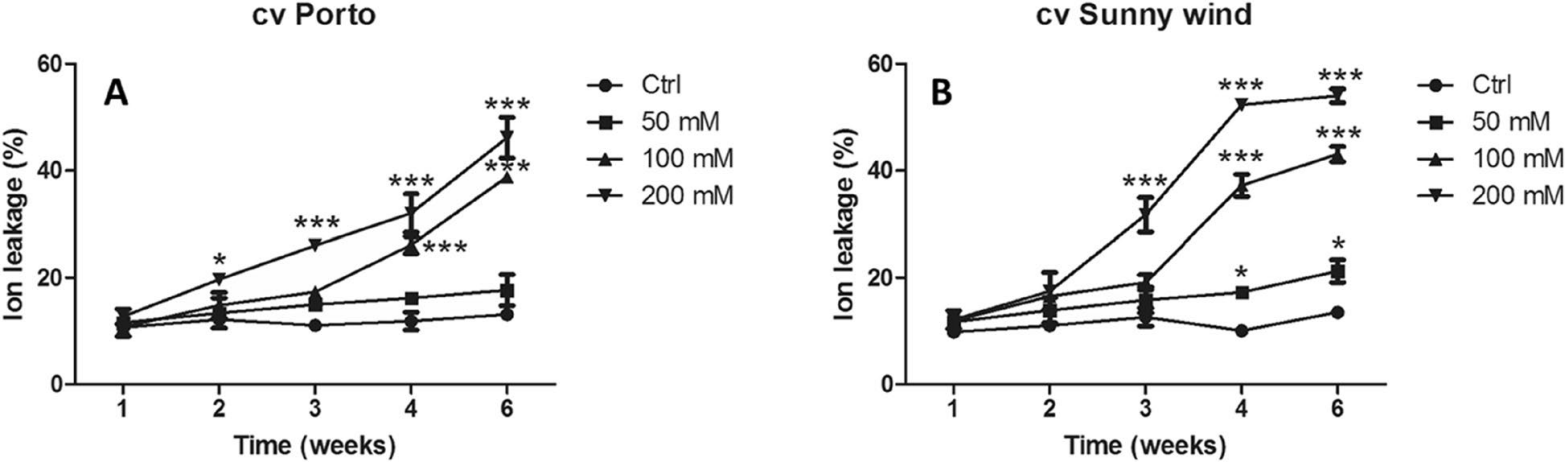
Ion leakage expressed as percentage in plants exposed to 0, 50, 100 or 200 mM NaCl. Data are shown as mean with at least 5 independent biological replicates and error bars indicate standard error. Two-way ANOVA were carried out and comparison among means values were separated using the Bonferroni multiple comparison test (*P* < 0.05). Significant differences are indicated by asterisks (* *P* < 0.05, ** *P* < 0.01, *** *P* < 0.001) of ion leakage (%) after different saline irrigation in cv “Porto” (**A**) vs cv “Sunny wind” (**B**)

Flowers from ‘Sunny wind’ plants showed a slightly reduction of flower fresh weight, -65% at 200 mM NaCl, while ‘Porto’ flowers showed a lower reduction compared to ‘Porto’ with value of 59% at 200 mM (Table 1). Flower weight production decreased gradually with increasing irrigation water salinity in both cultivars progressively declined under saline conditions (Fig. 1, Table 1). After 4 weeks from the beginning of 200 mM NaCl treatments, salt stress negatively affected flower production in ‘Sunny wind’ cultivar, since the plants did not produce more flowers, (Table 1). After six weeks of salinity stress at 200 mM NaCl almost 25% of ‘Sunny wind’ plants were completely died, while ‘Porto’ did not show any dead plants (Supplementary Table S1).

**Table 1:** Flower weight in ‘Porto’ and ‘Sunny wind’ exposed to 0, 50, 100 or 200 mM salinity. Data are shown as means with at least 10 independent biological replicates, and error bars indicate the standard error. Data were subjected to one-way ANOVA and differences among means were determined using Tukey’s post-test

Cultivar				
Treatment	**Porto**		**Sunny wind**	
**Ctrl**	6.10	± 0.24^a^	4.56	± 0.05^a^
**50 mM**	4.73	± 0.30^b^	3.42	± 0.27^b^
**100 mM**	3.72	± 0.29^c^	2.99	± 0.23^c^
**200 mM**	2.35	± 0.27^d^	1.64	± 0.32^d^

Different letters indicate statistical differences for *p* < 0.05

#### Ion leakage measurements

Membrane integrity in stressed plants was assessed by measuring ion leakage. The ‘Porto’ cultivar did not show any significant ion leakage increase under 50 mM NaCl treatment throughout the experimental period (Fig. 1 A), whereas 200 mM NaCl induced a significant increase 2 weeks after the beginning of the experiment (Fig. 1B). The ‘Sunny wind’ showed higher ion leakage (%) compared to cv ‘Porto’ after four and six weeks exposure to 100 mM or 200 mM NaCl treatments. ‘Sunny wind’ after six weeks at the highest salinity conditions showed an ion leakage of 55–58%, while in the ‘Porto’ the higher values were of 42–50% (Fig. 1 A, B).

The ‘Sunny wind’ cultivar showed a significant increase of ion losses after three weeks of exposure to salinity in plants grown under 200 mM NaCl. After four weeks, plants exposed to all salinity concentrations (50–200 mM) showed higher ion leakage (Fig. 1B).

#### Chlorophyll a fluorenscence measurements

**Fig. 2:**
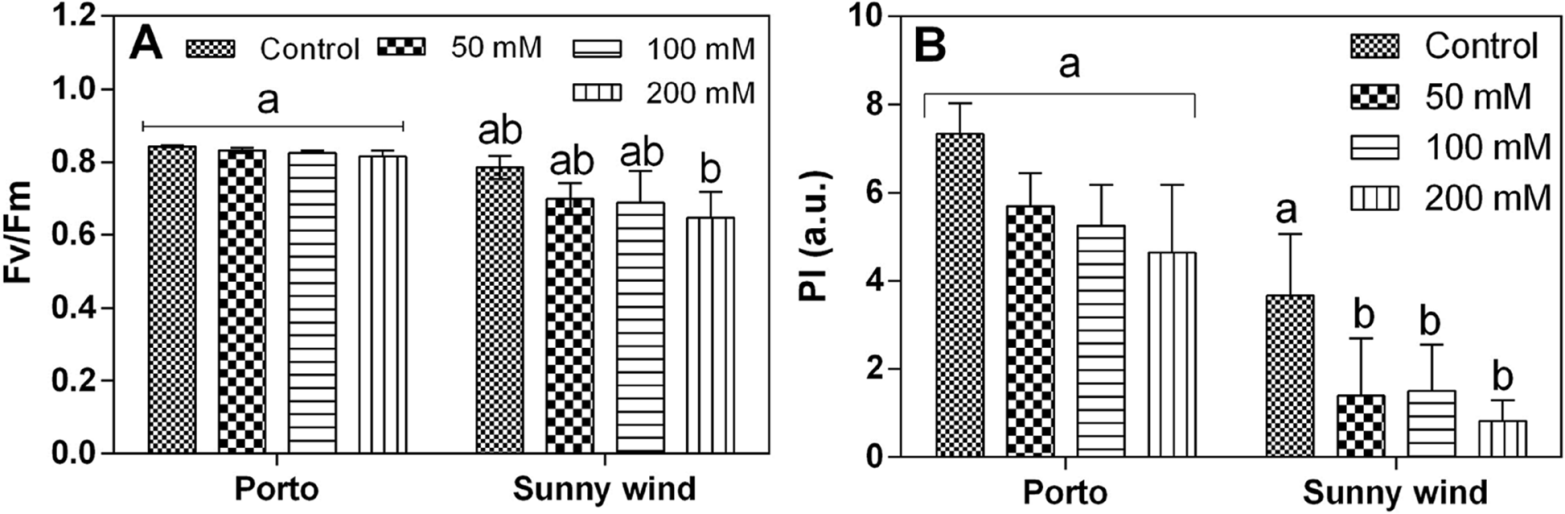
Chlorophyll a fluorescence parameters, Fv/Fm **A**) and performance index (PI), **B**) in ‘Porto’ and ‘Sunny wind’ plants exposed to 0, 50, 100 or 200 mM NaCl. Data are shown as mean with at least 5 independent biological replicates and error bars indicate standard error. Data were subjected to two-way ANOVA and differences among means were determined using Tukey’s post-test. Different letters indicate statistical differences for *p* < 0.05

The chlorophyll a fluorescence parameters measured after four weeks of salinity treatments demonstrated that the Porto cultivar did not show changes in the maximum quantum efficiency of photosystem II (Fv/Fm ratio), while Sunny wind cultivar shoer a progressive decline by increasing the salinity concentrations (Fig. 2A). The performance index (PI) calculated from chlorophyll a fluorescence data showed that ‘Porto’ had higher values compared to ‘Sunny wind’. These data suggest that leaf functionality was higher in ‘Porto’ and was retained in the different salinity stress conditions (Fig. 2B).

#### Abscisic acid content and proline concentrations

**Fig. 3:**
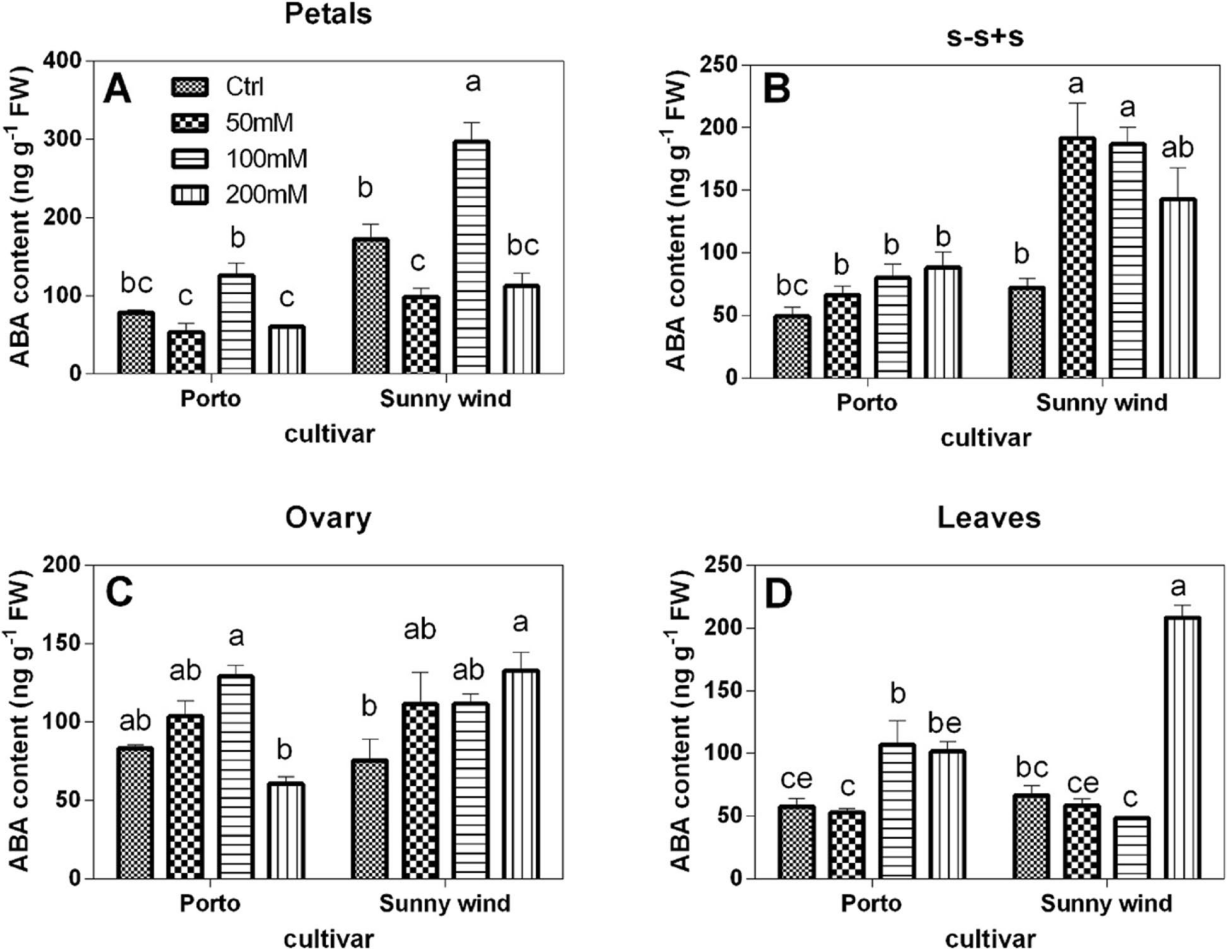
Abscisic acid (ABA) concentration (ng g.^−1^ FW) in petals (**A**), s–s + s (**B**), ovary (**C**), and leaves (**D**) in cv. Porto or cv. Sunny wind Hibiscus plants after 4 weeks of salinity exposure. Data are shown as mean with at least 5 independent biological replicates and error bars indicate standard error. Data were subjected to two-way ANOVA and differences among means were determined using Tukey’s post-test. Different letters indicate statistical differences for *p* < 0.05

The ABA concentration was higher in petals and ovary of flowers in both cultivars and between them the ‘Sunny wind’ showed double values in petals compared to Porto. The ABA content in petals showed an average of 90 and 190 ng g^−1^ FW in ‘Porto’ and ‘Sunny wind’, respectively (Fig. 3). Ovary showed an increase of ABA content in a concentration-dependent manner, except in ‘Porto’ that at 200 mM NaCl showed ABA levels similar to those observed in control plants. Increasing salinity did not affect ABA content in s–s + s organs of ‘Porto’,, while doubled in ‘Sunny wind’ from 90 ng g-1 FW to 180–195 ng g^−1^ FW in plants when salinity reached 50 to 200 mM. Leaves showed lower ABA concentrations, which did not linearly increase with salinity. In the ovary of ‘Porto’cv. an ABA increase was observed in 100 or 200 mM NaCl, whereas in’Sunny wind’ it was only observed under the highest salinity exposure (200 mM NaCl) with a concentration above 200 ng g^−1^ FW.

**Fig. 4:**
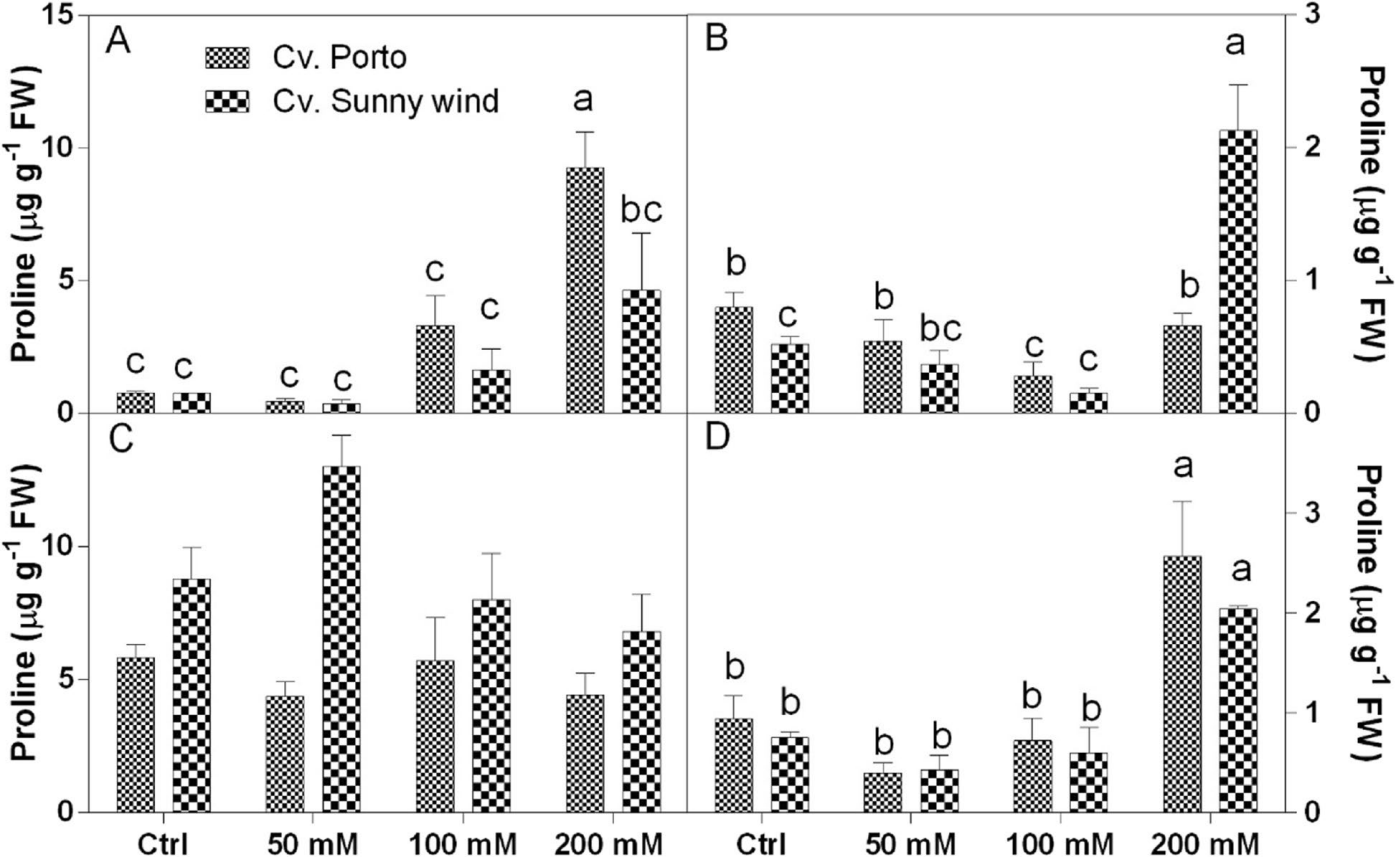
Proline concertation in leaves, **A**) petals **B**), s–s + s **C**), and ovary **D**) of *Hiscus rosa-sinensis* L. cultivars, ‘Porto’ or ‘Sunny wind’. Data are shown as mean with at least 5 independent biological replicates and error bars indicate standard error. Data were subjected to two-way ANOVA and differences among means were determined using Tukey’s post-test. Different letters indicate statistical differences for *p* < 0.05

Proline concentrations were analysed in different flower organs (Fig. 4). Results revealed that higher values were observed in s–s + s (Fig. 4C) of ‘Sunny wind’ compared to ‘Porto’. In the leaves, both cultivars showed a significant increase of proline at 100 and 200 mM NaCl (Fig. 4A). At these salinity concentration ‘Porto’ plants had higher proline concentration than ‘Sunny wind’. In ‘Porto’ the increase of proline in plants treated with 200 mM NaCl was 18–20 times higher compared to those observed in the untreated ones. In s–s + s organs of ‘Porto’ the proline concentration did not significantly change under different salinity conditions, with values ranging from 4 to 6 µg g^−1^ FW. In ‘Sunny wind’ the proline increased in s–s + s tissue under 50 mM NaCl. Proline concentration in the ovary did not change between cultivars, but a significant increase was observed under 200 mM NaCl exposure. Under these salinity conditions, proline levels increased 2–5-times, reaching 2–2.5 ng g^−1^ FW. At 200 mM in both cultivar the proline increased, but in “Sunny wind” the concentration was 4–5 time higher than the control (Fig. 4D).

**Fig. 5:**
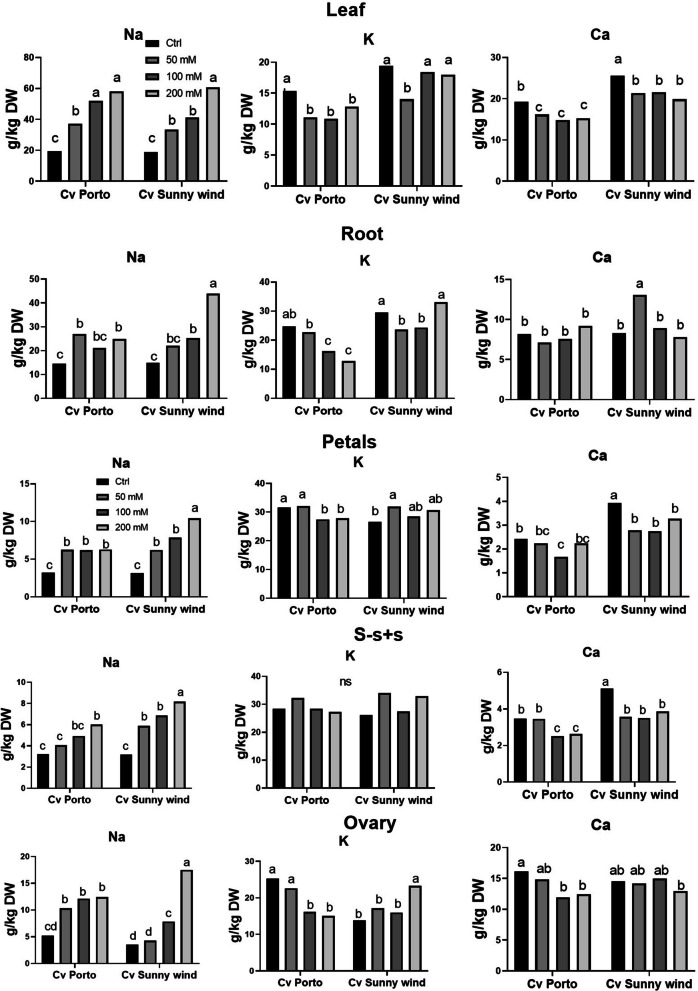
Determination of sodium (Na), potassium (K), and calcium (Ca) in leaves, roots, petals, s–s + s and ovary of ‘Porto’ and ‘Sunny wind’ cultivars after four weeks of salinity exposure (50, 100, or 200 mM.) Data were subjected to two-way ANOVA and differences among means were determined using Tukey’s post-test. Different letters indicate statistical differences for *p* < 0.05

Sodium (Na), potassium (K), and calcium (Ca) were determined in leaves and different plant organs. Na in leaves increased by increasing the salinity concentration in both cultivars (Fig. 5). Both cultivars exhibited a similar trend in Na + concentration in leaves under salinity treatments, but ‘Sunny wind’ showed a higher increase in Na + concentration in root. In flower organs, the Na did not increase in ‘Porto’ with the increase of salinity level. In ‘Sunny wind’, instead, the Na increased following the salinity stress in all flower organs and ovary was the flower organ with the highest fold increase.

The K declined with the increase of salinity, especially in ‘Porto’ leaves and roots. In ‘Sunny wind’ the K reduction was higher at lower salinity conditions, such as 50 mM NaCl.. In the ovary, the K concentration declined in ‘Porto’ by increasing the salinity levels, while opposite behaviour was observed in Sunny wind cultivar. In petals, the K concentration showed the same trend as that observed for the ovary for both cultivars. The Ca concentration was lower than control almost in all conditions, except in root and ovary of ‘Sunny wind’ that showed a concentration above the control at 50 and 100 mM NaCl, respectively (Fig. 5).

### Transcriptional profiles

#### Differential expressed genes

**Fig. 6:**
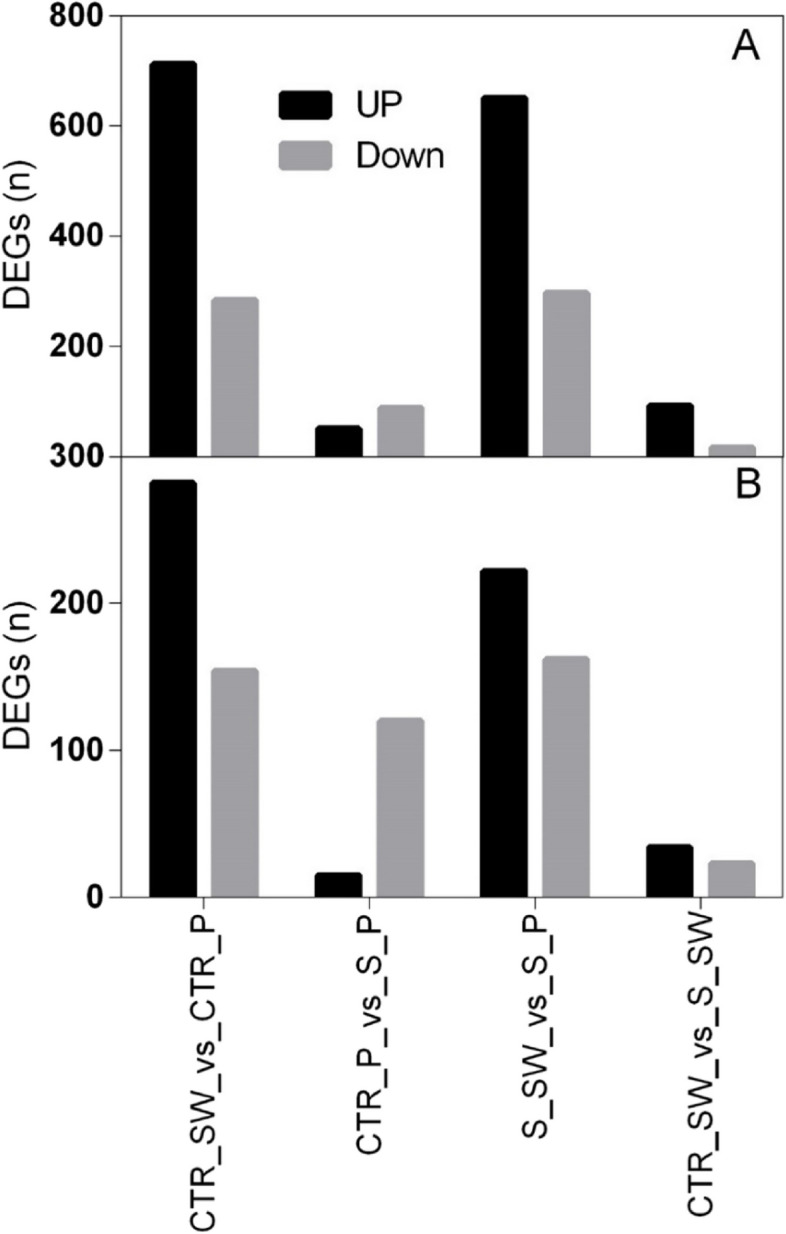
Differential expressed genes (DEGs) up-or downregulated in hibiscus flower organs of two cultivars, ‘Porto’ and ‘Sweet wind”. **A** DEGS in petals, **B** DEGs in Ovary + style-stigma + stamens (SO). Treatments compared are the following: CRT_SW (‘Sweet wind’ control), CTR_P (‘Porto’ control), S_SW (‘Sweet wind’ exposed to salinity 100 mM NaCl for four weeks), and S_P (‘Porto’ exposed to salinity 100 mM NaCl for four weeks)

To unravel the transcription-based regulatory network occurring in the flowers of two Hibiscus rosa-sinensis L. cultivars (Porto and Sunny wind) treated with 100 mM NaCl for four weeks, an Agilent custom gene expression microarray previously based on 454 GS-FLX sequencing of Hibiscus flower tissues (Trivellini et al. 2016a, b) was performed. Transcriptome analysis was performed on isolated flower tissues, petals (Pet) and style-stigma plus stamen and ovary, (SO). The following comparisons were performed: CRT_SW (‘Sweet wind’ control), CTR_P (‘Porto’ control), S_SW (‘Sweet wind’ exposed to salinity 100 mM NaCl for four weeks), and S_P (‘Porto’ exposed to salinity 100 mM NaCl for four weeks). Profiling indicated that a large number of transcripts were differentially expressed in ‘Porto’ and ‘Sunny wind’ cultivars under control condition: 996 differential expressed genes (DEGs) in petals and 436 in ovary + style and stigma + stamens (SO). Upon four weeks salt stress, 948 genes in petals (Fig. 6A) and 384 in SO (Fig. 6B) showed differential expression in ‘Porto’ and ‘Sunny wind’ cultivars showing a change of twofold or more with a significant *P* value (*P* ≤ 0.05) (Supplementary Table S2).

**Fig. 7:**
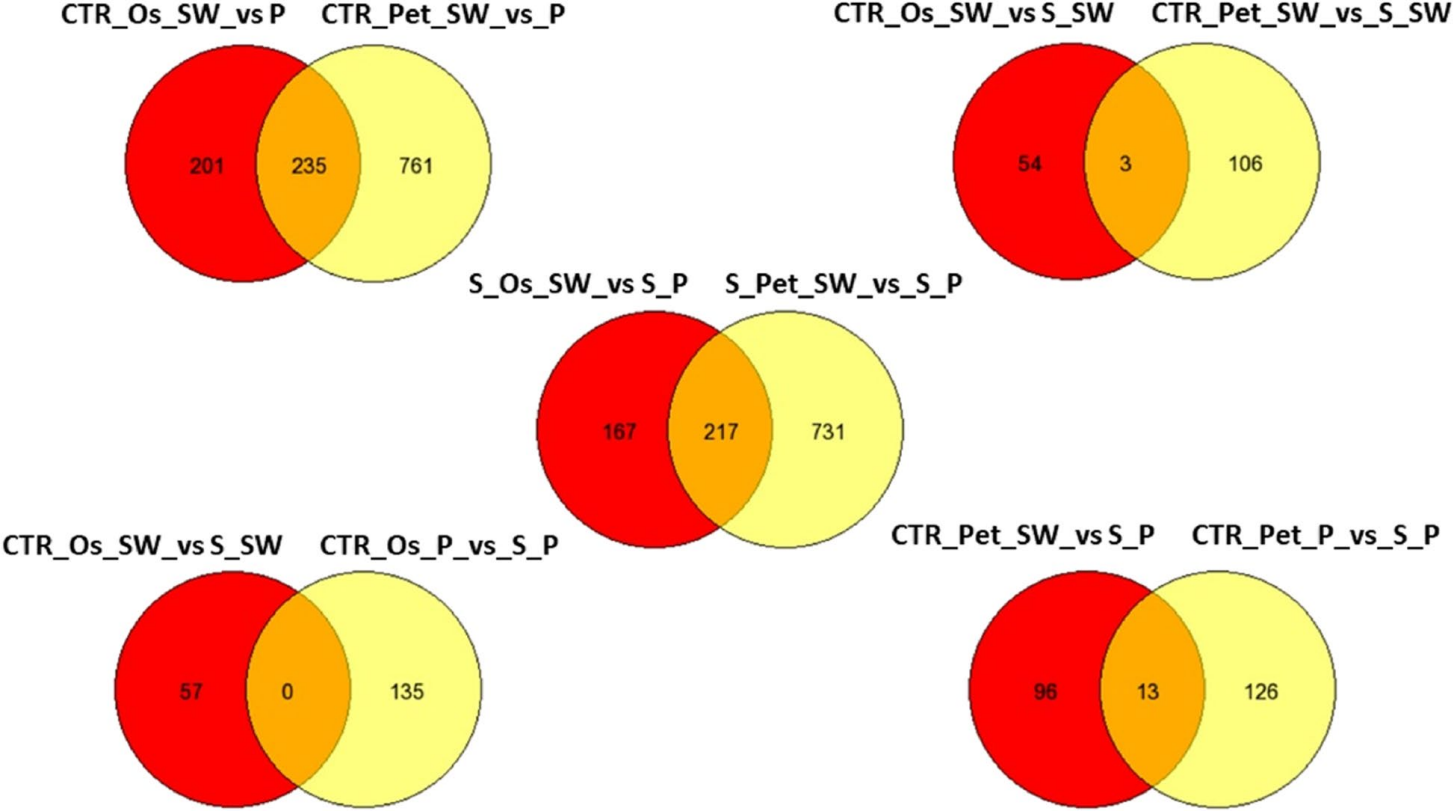
Venn diagram of the differentially expressed genes among cultivars, salinity, and flower organs. The detailed information of genes in common or with different responses can be found in Supplementary Table S2

Analysing the DEGs between transcriptional conditions (Fig. 7), revealed that 235 DEGs were in common between SO and petals of the two cultivars under no stress conditions. Of the commonly regulated genes under control versus salinity stress in both cultivars, three genes were identified in OS versus petals. These encoded for protein involved in the photosynthetic pathways (XP_002303160.1| light-harvesting complex II protein Lhcb6) for chloroplast or photosystem I (XP_002516419.1| Photosystem I reaction center subunit III, chloroplast precursor). Upon salt stress, 217 commonly regulated genes were identified in both petals and SO of ‘Porto’ and ‘Sunny wind’ flower tissues. It is interesting to note that 731 DEGs were specifically found in petals and 167 in Os + s–s + s (Fig. 7). No DEGs in common were found in control versus salinity in ovary between cultivars, while in petals there were found 10 DEGs (Table 2).

**Table 2:** Common DEGs (10) significantly expressed under salinity conditions in both cultivars. Values are mean of LogFC

Best hit to nr and Accession number	GO Molecular Function	Sunny wind	Porto
AAF04851.1|AF195796_1 putative alcohol dehydrogenase	GO:0000166 nucleotide binding | GO:0008270 zinc ion binding | GO:0004022 alcohol dehydrogenase (NAD) activity	4.03	3.82
ACD56606.1| alcohol dehydrogenase A	-	3.73	3.93
YP_720000.1| coat protein	-	2.51	2.45
ABL67651.1| putative auxin-repressed/dormancy-associated protein	-	2.28	2.00
Unknown		2.16	2.13
Unknown		2.15	2.08
ABW89470.1| low molecular weight heat shock protein	-	-2.27	-2.56
XP_002300789.1| cytochrome P450	GO:0009055 electron carrier activity | GO:0020037 heme binding | GO:0070330 aromatase activity	-2.31	-2.08
CBI33176.3| unnamed protein product	GO:0016747 transferase activity, transferring acyl groups other than amino-acyl groups	-2.59	-3.99
CAD87535.1| putative xyloglucan endotransglycosylase	GO:0004553 hydrolase activity, hydrolyzing O-glycosyl compounds | GO:0016762 xyloglucan:xyloglucosyl transferase activity	-2.60	-2.47

**Fig. 8:**
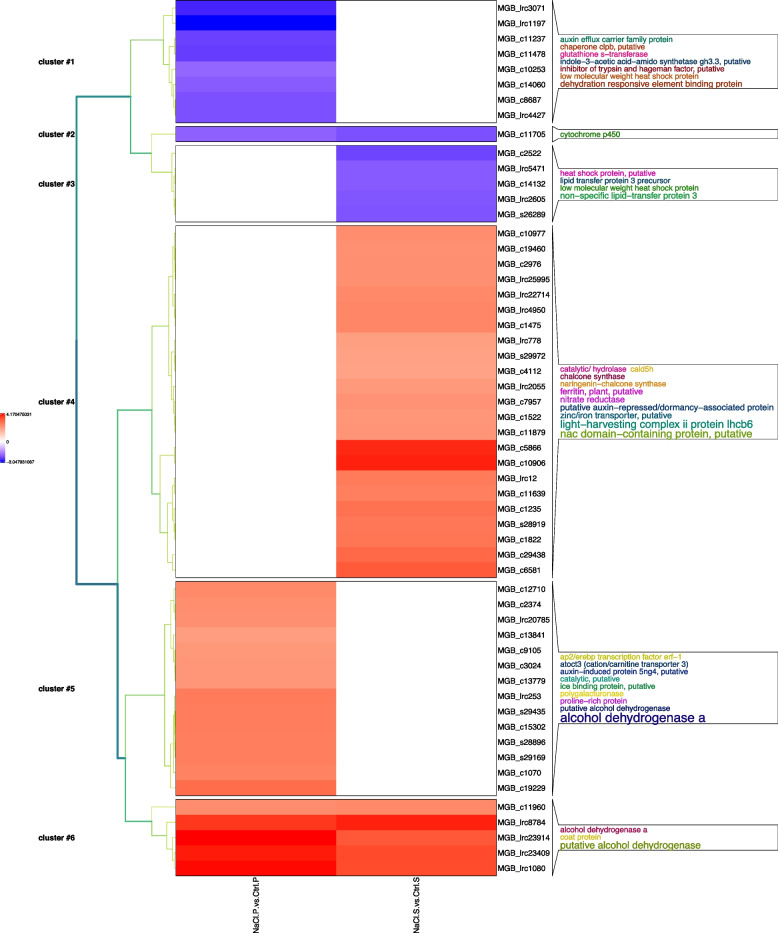
Cluster analysis of annotated transcript significantly regulated in ‘Porto’ (P) and ‘Sunny wind’ (S) petals after four weeks of salt treatment (100 mM). The log-2 ratio values of genes significantly regulated by salt stress were used for hierarchical cluster analysis. The red represents up-regulated genes; the blue represents down-regulated genes; the black represents un-regulated genes

**Fig. 9:**
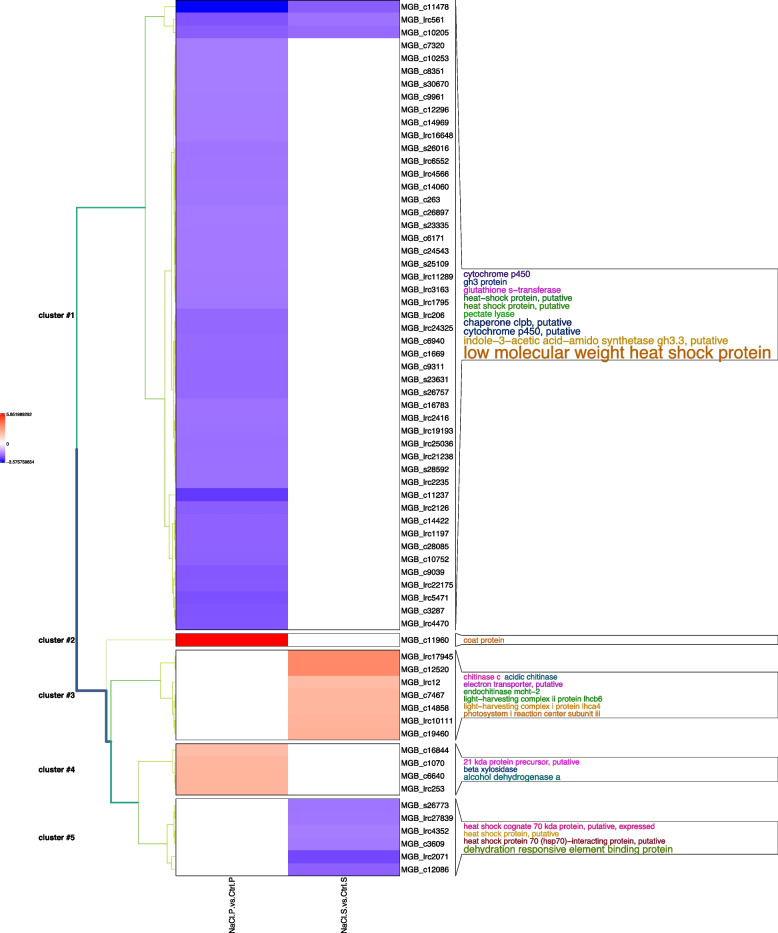
Cluster analysis of annotated transcript significantly regulated in ‘Porto’(P) and ‘Sunny wind’ (S) SO after four weeks of salt treatment (100 mM). The log-2 ratio values of genes significantly regulated by salt stress were used for hierarchical cluster analysis. The red represents up-regulated genes; the blue represents down-regulated genes; the black represents un-regulated genes

Since ‘Porto’ cv is more tolerant than ‘Sunny wind’ a hierarchical cluster analysis of differentially regulated transcript with known function following Blastx NCBI Nr annotation in petal (Fig. 8) and in SO (Fig. 9) were performed. Six gene clusters were identified in petal tissues of ‘Porto’ (P) and ‘Sunny wind’ (S) using a transcript ratio of > 2 as a filter (Fig. 8).

Cluster 1 contained 8 transcripts showing downregulation by -2 to -fourfold in petals of ‘Porto’ cv. Among them were genes encoding heat shock proteins (HSP), Indole-3-acetic acid-amido synthetase (GH3.3), auxin efflux carrier protein, chaperone clpb and glutathione S-transferase. Two genes encoding dehydration responsive element binding protein (DREB) also showed down-regulation by -2.30- to 2.83-fold.

Only one gene (Cluster 2) was shown to be specifically downregulated in both ‘Porto’ and ‘Sunny wind’ petals. It encodes a Cytochrome P450 gene belonging to a superfamily of enzymes containing heme as a cofactor that mostly, but not exclusively, function as monooxygenases. The cluster 3 consisted of genes down-regulated only in ‘Sunny wind’. Among them, there were two genes encoding small heat shock protein and other three genes encoding lipid transfer protein 3. Cluster 4 includes 23 transcripts that were significantly up-regulated by four weeks of salt stress only in ‘Sunny wind’ petals. Within this cluster, the largest functional category of identified genes was the light-harvesting complex II protein Lhcb6 containing 5 transcripts. The second main functional category of identified salt responsive genes in ‘Sunny wind’ petals was transcription regulator category, which include four transcripts encoding transcription factors (TFs) such as NAC transcription factor. Further examination of the genes classified into cluster 4 identified two genes encoding zinc/iron transporters and two genes encoding ferritin. Interestingly, two nitrate reductase and two chalcone synthase genes were also present in cluster four, along with two putative auxin-repressed/dormancy-associated proteins and coniferaldehyde 5-hydroxylase (CAld5H).

The up-regulated genes induced only in ‘Porto’ petals of cluster 5 were dominated by transcripts encoding alcohol dehydrogenase genes (6 out of 14). This cluster also includes genes encoding a proline-rich protein, auxin-induced protein 5ng4, cation/carnitine transporter 3, a polygalacturonase and AP2/EREBP transcription factor ERF1.

Cluster six includes a relatively small number of genes up-regulated in both ‘Porto’ and ‘Sunny wind’ petals. A comparison of the fold induction of these genes in both genotypes revealed that the majority of the genes were induced at similar magnitude and encoding four alcohol dehydrogenase genes and one coat protein.

In SO tissues of ‘Porto’ and ‘Sunny wind’, five gene clusters were identified in petal tissues using a transcript ratio of > 2 as a filter (Fig. 9).

Of the 67 differentially regulated transcripts in SO tissues of ‘Porto’ and ‘Sunny wind’ plants under salinity stress (Supplementary Table S1), 49 were clustered into Cluster 1, which is the largest cluster, containing 89.10% of all downregulated regulated annotated transcripts (Fig. 9, Supplementary Table S2). In Cluster 1, the largest identified functional category comprised the response to stress which had 24 members and comprised 49.00% of cluster 1. This category included genes encoding 18 heat shock proteins, three genes encoding chaperone clpb, and two genes encoding glutathione S-transferase which could act as molecular chaperones which perform under stress situations predominantly in SO tissue of ‘Porto’ cultivar. Interestingly, six Indole-3-acetic acid-amido synthetase GH3 transcripts, cytochrome P45 and two pectate lyases were also detected in cluster 1 (Fig. 9).

Cluster 2 contained only a coat protein strongly up-regulated (5.8 fold) in SO of ‘Porto’ cultivar. The third cluster consisted of genes up-regulated only in ‘Sunny wind’ SO tissues. Among them, there were three genes the encoding chitinase, two genes encoding light-harvesting complex and one a photosystem I reaction center. The up-regulated transcripts in SO of ‘Porto’ cultivars were all included in cluster 2 and 4. Within cluster 4, two alcohol dehydrogenase genes and a beta xilosidase were comprised. The transcript downregulated only in SO of ‘Sunny wind’ cultivar were included in cluster 5. This cluster was entirely represented by the functional category stress response which comprised 6 members with three transcripts encoding dehydration responsive element binding protein (DRE) and three transcripts encoding heat shock protein.

## Discussion

In ornamental plants, salinity stress reduces growth, flower size, flower turnover, and visual quality (Toscano et al. 2020). It is well known that salinity reduces photosynthesis and carbohydrate levels, which are useful for flower production and development. The consequence of this is a reduction in biomass accumulation, as observed in plants and flowers. These results were also observed and confirmed in two *Hibiscus syriacus* L. cultivars exposed to salinity up to 0–200 mM NaCl (Lu et al. 2022). The reduction in leaf functionality and leaf gas exchange under saline conditions can also be evaluated using chlorophyll fluorescence. This non-destructive method has been used to screen and select ornamental plants that are tolerant to salinity (Ferrante et al. 2011; Farieri et al. 2016). Plants absorb and use light under optimal conditions; when plants are exposed to abiotic stresses, including salinity impairment of the photosynthetic machinery gradually decreases the light use efficiency. Chlorophyll *a* fluorescence and related parameters allow for the evaluation of plant health status and stress conditions (Toscano et al. 2020).

Salinity induces ROS accumulation, which is primarily responsible for the membrane degradation. Ion leakage is closely correlated with membrane integrity (Hatsugai and Katagiri 2018). Results indicate that ‘Sunny wind’ shows a notable sensitivity to salinity compared to ‘Porto’, resulting in a loss of membrane integrity at higher salt concentrations and after longer exposure. Another biochemical indicator of salinity stress is ABA, which, along with its signalling pathway, plays an important role in plant adaptation to stress. ABA is a plant hormone that regulates stomatal opening, and is largely influenced by water availability. However, it also plays a role in the osmotic regulation during salinity stress. In particular, a previous study reported that the expression of *salt overly sensitive 1 (SOS1*) and *high affinity K transport 1* genes are probably under ABA signaling network (Osakabe et al. 2014). The modulation of endogenous ABA content in ‘Porto’, suggests that this cultivar is more salt tolerant than ‘Sunny wind’. The ‘Porto’ cultivar showed a significant increase of ABA in leaves that may contribute to plant tolerance. Changes in ABA in flower organs were particularly high in petals of ‘Sunny wind’.

Under saline conditions, an increase in proline levels can be used as a stress indicator. Proline is a non-protein amino acid that is also a soluble osmolyte that increases during stress conditions such as cold, drought, and salinity (Boscaiu et al. 2013; Ghosh et al. 2022). Proline protects plant cells against oxidative stress by removing ROS (Toscano et al. 2023). The higher increase of proline concentration in ‘Porto’ leaves at the highest salinity condition could be associated to the tolerance to salt stress and explain the survival and the ornamental quality retain. Among flower organs, a proline increase was observed at the highest salt concentration (200 mM NaCl), whereas significant differences between cultivars were only observed in the leaves and petals. Lower proline concentrations are associated with higher tolerance to salinity. An experiment performed on chrysanthemum showed that heterografting enhanced salinity tolerance and lowered the proline concentration (Li et al. 2022). Treatment with NaCl in purslane increased proline concentration, which is considered to play a key role in the salinity tolerance mechanisms of this ornamental plant (Yazici et al. 2007).

The salinity induced by Na directly effects ion uptake. Previous studies have demonstrated that mineral content varies during development in different hibiscus flower organs (Trivellini et al. 2011b). Under saline conditions, ion accumulation is influenced by Na concentration, which affects several ion uptakes, especially K. In *Hibiscus moscheutos,* seedlings exposed to 200 mM NaCl showed an increase in Na^+^ and a dramatic decrease in K^+^ in the leaves (Feng et al. 2021). In different plants, it has been observed that an increase in Na^+^ reduces K^+^ uptake, since they compete for the same transporter (Shabala and Cuin 2008). In ‘Porto’ the increase of Na uptake seems to be correlated with K in leaves, while no changes were found in flower organs that could be ameliorated by Ca. It is well known that Ca regulates the Na translocation by acting on *SOS* genes (Toscano et al. 2023).

In order to compare the salt-transcriptional response of ‘Porto’ and ‘Sunny wind’ cultivars on flower growth, a floral tissue-specific microarray analysis was performed treating the plants with 100 mM of NaCl since at this concentration for both cultivars all of the plants still continue to flowering (to produce flowers). Gene transcripts that are present in floral tissue of a particular cultivar and gene transcripts that are present in higher abundance in these tissues are valuable for understanding the biological and metabolic processes during salinity tolerance mechanisms. In general, different transcriptional profiles were obtained in the more tolerant ‘Porto’ cultivar and the moderately sensitive ‘Sunny wind’ under salt-stressed and unstressed conditions. In response to salt stress, the differential regulation of a relatively larger number of floral specific genes between the cultivars was observed and these findings suggested that the degree of salt tolerance may be directly linked to the extent of transcriptome modification by salt stress. Among the common responses between the different floral tissues in ‘Porto’ and ‘Sunny wind’ cultivars, an interesting downregulated gene in petals and SO was the DREB that has been associated with salinity tolerance in different plant species. Functional studies in Arabidopsis demonstrated that overexpression of *GhDREB1* increased sensitivity to high salinity (Dong et al. 2010). DREB gene has been supposed to act as transcriptional repressor for DRE-mediated gene expression (Huang and Liu 2006). However, in tomato plants exposed to salinity the expression of SlDREB2 and SlDRB3 showed an increase during the first 12 h and then the trend declined (Islam and Wang 2009; Hichri et al. 2016). These findings remark the importance of DREB, but their function could be associated with early adaptation responses to salinity. Among the upregulated genes there were coat protein (COP) genes that seems to be associated to salt tolerance. Functional analysis using mutants with loss function of COP genes showed higher sensitivity to salinity, demonstrating the importance of these genes under salt stress (Sánchez-Simarro et al. 2020). Another highly expressed gene was the alcohol dehydrogenase (ADH) gene which has been found expressed in both cultivars but with different abundance. In detail, in this study, five ADH genes commonly regulated in petals of both cultivar and eight only in ‘Porto’ were identified. The incorporation of ADH genes from *Synechocystis sp*. PCC 690 in tobacco plants resulted in transgenic *N. benthamiana* plants with enhanced salt tolerance (Yi et al. 2017). Moreover, transgenic lines grown under 300 mM NaCl also showed higher expression level of stress-related genes such as *DREB2A*, responsive to desiccation 29 (RD29), and HSP17.6 (Yi et al. 2017). The higher number of ADH transcripts found in ‘Porto’ floral tissues may be involved in defence responses when challenged by abiotic stress for a stress-induced signal transduction leading to the activation of salt-tolerance-related responses.

No common genes differentially regulated were found between the cultivars (Figs. 7 and 8). Focusing on salt tolerance of ‘Porto”, in this study an *APETALA2/ETHYLENE RESPONSIVE FACTOR (AP2/ERF)* transcription factor *ERF1* was upregulated in petals. This family of transcription factor has emerged as key regulators of various stress responses, to help activate plant hormone signaling pathways that in turn mediate plant survival under stress conditions (Xie et al. 2019). In Arabidopsis, expression of *ERF1* was rapidly and transiently induced by salt and dehydration treatments in an ABA-independent manner, and the overexpressing plants were more tolerant to drought, salt, and even heat stress (Cheng et al. 2013).

Plant cell wall integrity is associated with abiotic stress tolerance, as significant modifications to cell wall composition have been linked to an improved plant fitness against adverse environmental conditions (Zhang et al. 2021). Hypersensitivity to salinity stress can be a consequence of defects in cell wall biosynthesis as *Salt Overly Sensitive5 (SOS5)* gene which encodes a putative cell surface adhesion protein and is required for normal cell expansion (Shi et al. 2003). Moreover, in Arabidopsis, a cell wall structural protein, a proline rich protein (PRP3), has been reported to promote seed germination and maintain cell wall integrity under cold and high-salinity stress condition (Qin et al. 2013). In ‘Porto’ floral tissues exposed to salt, three cell wall associated genes, a proline-rich protein, a polygalacturonase and a pectate lyase were found to be differentially regulated in floral tissues and may be function in cell wall remodelling in the context of tissue growth and development when facing salinity stress. Among the downregulated genes specifically regulated in ‘Porto’ cultivars, two transcripts in SO tissue and one transcript in petals encoding an indole-3-acetic acid-amido synthetase GH3.3 were detected. In the moss *Physcomitrium patens*, loss of the auxin-conjugating GH3 enzymes results in tolerance to high salt concentrations (Koochak and Ludwig-Muller 2021). Similarly, in Arabidopsis the disruption through the multiple knockout of GH3s or GH3-like protein genes might modulate plant tolerance to salt stress by redundant auxin conjugation (Casanova‐Sáez et al, 2022). Overall, the above results leading to lower free IAA and the transcriptional modulation of endogenous IAA concentrations through the downregulation of IAA inactivation target genes, GH3, in ‘Porto’ cultivars, suggest a connection between auxin biosynthesis and salt stress response. Other dowregulated gene was NCED. The NCED is a key regulator of ABA biosynthesis through carotenoids degradation. The lower expression could be associated with lower ABA concentration that could modulate the salt responses of different organs. In general, the overexpression of NCED has been associated to higher ABA and ROS with positive effect on salinity tolerance (Zhang et al. 2008).

## Conclusion

Under the present investigated conditions and analytical method, our results clearly indicated that salt tolerance of ‘Porto’ cultivar can be the manifestation of several physiological processes. This tolerance was appreciated at morphological, physiological, biochemical, and molecular level. At biochemical level, the proline seems to be the major parameters that highlighted the differences between cultivars especially in leaves. Chlorophyll *a* fluorescence has been confirmed to be a good tool to discriminate salt tolerance non-destructively. Comparing of transcriptomes of salt-tolerant ‘Porto’ and –sensitive ‘Sunny wind’ cultivars indicated the different transcriptome might be important reasons of the tolerance. Porto plants had enhanced expression of many alcohol dehydrogenase genes, a coat protein, an *AP2-ERF1* transcription factor, members involved in cell wall structure and modification and downregulation of genes play important roles in hormone biosynthesis and inactivation such as *NCED* and GH3 respectively, which likely all contributed incrementally to explain the increased salt tolerance. These findings may greatly contribute to a better understanding of hibiscus tolerance to salt stress and provide more candidate genes for engineering salt-tolerant ornamental crops.

## Materials and methods

### Plant materials and growth conditions

Plants of *Hibiscus rosa-sinensis* L. Porto and Sunny wind cultivars were used for the salinity experiments and transcriptome analysis. Plants were grown in 3 L plastic pots containing peat/pumice (1:1, v/v) in an greenhouse belonging to the University of Pisa (43◦42.343N and 10◦25.280E). Treatments with saline irrigation water (50, 100 and 200 mM NaCl) or irrigation water (control) were applied once every day for 60 s using an automatic system, for a total of 6 weeks.

Fertilization was performed directly into the irrigation water. For each treatment (50 mM, 100 mM, 200 mM and the control) were used about 30 plants/cultivars.

### Chlorophyll a fluorescence

It is a non-destructive, fast and easy system to perform measurement. Chlorophyll a fluorescence and the parameters derived from it have often been used for studying plant stress in a wide range of stressful conditions such as useful tool for identifying tolerant species (Ferrante et al. 2011). Chlorophyll *a* fluorescence transients were determined on dark adapted leaves kept for 30 min at room temperature, using a portable Handy PEA (Hansatech, UK). Measurements were taken of the leaf surface (4 mm diameter) exposed to an excitation light intensity (ultrabright red LEDs with a peak at 650 nm) of 3000 μmol m^−2^ s^−1^ (600 Wm^−2^) emitted by three diodes. Leaf fluorescence detection was measured by a fast response PIN photodiode with a RG9 long pass filter (Hansatech, technical manual). The parameters measured were Fo, Fm, and Fv/Fm. JIP analysis was performed to determine the performance index (PI).

### Membrane integrity

Leaf injury is another screening parameter for salinity tolerance and can be measured by ion leakage.

For electrolyte leakage, the leaves were sampled every 7 days until the end of the experiment. Ten leaf disks were incubated by shaking in 5 mL of distilled water for 4 h at room temperature. After the incubation, the conductivity in the solution was determined by using an HI8733 conductivity meter (Hanna Instruments). Then, the samples were autoclaved and cooled, and the conductivity was read again in the solutions. Ion leakage was expressed as a percentage of the total conductivity after autoclaving (Scotti and Thi, 1997).

### Mineral determinations

The petal, style-stigma plus stamen (s–s + s), and ovaries and leaves were isolated from harvested flowers and used for mineral analysis. Each plant tissue was grouped in three independent biological replicates of 20 flowers, dried for 3 days in a 70 °C oven, ground in a mortar with pestle to pass a 40-mesh screen, and stored at -20 °C for later analysis. Fresh weight (FW) was taken immediately before drying. Dry weight (DW) was measured after the tissues were ground to a powder. The oven-dried-ground samples were wet digested in a mixture of nitric–perchloric acids (HNO_3_: HClO_4_, 5: 2 v/v). Potassium, calcium, sodium concentrations in the wet digested samples were quantified by atomic absorption spectrophotometry (Varian Model Spectra-AA240 FS, Australia).

### Abscisic acid determination

Abscisic acid (ABA) was determined by an indirect ELISA based on the use of a DBPA1 monoclonal antibody raised against S( +)- ABA (Vernieri and others 1989). The ELISA was performed according to the method described by Walker- Simmons (1987), with minor modifications. Petals, styles-tigma plus stamen, and ovary samples (approximately 100 mg FW) were collected, weighed, frozen in liquid nitrogen, and then stored at -80 °C until the analysis. ABA was measured after extraction in distilled water (water:tissue ratio = 10:1 v/w) overnight at 4 °C. Plates were coated with ABA-40-BSA conjugate at 200 µl per well and incubated overnight at 4 °C, then washed three times with 75 mM PBS buffer, pH 7.0, containing 1 g L^−1^ BSA and 1 ml L^−1^ Tween 20, keeping the third washing solution for 30 min at 37 °C. Next, 100 µl ABA standard solution or sample and, afterwards, 100 µl DBPA1 solution (lyophilized cell culture medium diluted in a PBS buffer containing 10 g L^−1^ BSA and 0.5 ml L^−1^ Tween 20, at a final concentration of 50 µg ml^−1^) were added to each well and competition was allowed to occur at 37 °C for 30 min. Plates were then washed again as described above and 200 µl per well of a secondary antibody (alkaline phosphatase-conjugated rabbit anti-mouse (Sigma-Merck Italy) in a PBS buffer containing 10 g L^−1^ BSA and 0.5 ml L^−1^ Tween 20, at a final dilution of 1:2000) was added and incubated for 30 min at 37 °C. Plates were washed again and p-nitrophenyl phosphate at 200 µl per well was added and incubated for 30 min at 37 °C. Absorbance readings at 415 nm were obtained using a MDL 680 PerkinElmer microplate reader. For each treatment, five independent samples were assayed in triplicate.

### Proline level

Determination of free proline content was done according to Bates et al. (1973). Petals, styles-tigma plus stamen, and ovary samples (500 mg FW) were collected and homogenized in 3% (w/v) sulphosalycylic acid and homogenate filtered through filter paper. After addition of acid ninhydrin and glacial acetic acid, resulting mixture was heated at 100 ◦C for 1 h in water bath. Reaction was then stopped by using ice bath. The mixture was extracted with toluene, and the absorbance of fraction with toluene aspired from liquid phase was read at 520 nm. Proline concentration was determined using calibration curve and expressed as µmol proline g^−1^ FW.

### Transcriptome and transcriptional profile analysis

RNA isolation and double-strand cDNA preparation for 454 sequencing. Flowers of *H. rosa-sinensis* L. ‘Porto’ and ‘Sunny wind’ plants were used for the transcriptome sequencing. Flowers from plants grown for 4 weeks under 100 mM NaCl were harvested on the morning of flower opening, immediately dissected in petal, and style-stigma-stamen plus ovary and ground under liquid nitrogen before extraction of total RNA using the Spectrum Plant Total RNA Kit with on-column DNase-treatment (Sigma, Italy) according to the manufacturer’s instructions. RNA concentration and integrity were assessed with a NanoDrop N-1000 spectrophotometer (ThermoFisher Scientific) and standard formaldehyde agarose gel electrophoresis (Supplementary Fig. S1). First strand cDNA synthesis was performed using the SMART cDNA Synthesis Kit (Clontech Laboratories) and SuperScript III Reverse Transcriptase (Life Technologies).

Double-stranded cDNA was amplified by long-distance PCR using the Advantage® 2 PCR Enzyme System (Clontech Laboratories). Amplification was performed in a thermal cycler (Applied Biosystems) with the following PCR parameters: 95 °C for 1 min; 16 cycles of 95 °C for 15 s and 65 °C for 30 s; 68 °C for 6 min; and 4 °C for 45 min. The double-stranded cDNA was resolved in a 1.1% agarose gel containing ethidium bromide and the cDNA synthesis verified by visualization under UV light. Samples were sequenced using the 454 GS-FLX instrument according to the manufacturer’s instructions (Roche). Transcriptome data have been published in the GenBank database [PRJNA325155].

### Microarrays analysis: cDNA synthesis, labelling and hybridisation

Microarray chips were built using transcriptome sequences and chip production were performed by Agilent custom service. Total RNA was amplified using the Amino Allyl MessageAmp II aRNA Kit (Ambion) to obtain amino allyl antisense RNA (aaRNA), following the method developed by Eberwine and coworkers. Briefly, mRNA was reverse transcribed into single-stranded cDNA; after the second strand synthesis (in the second round of amplification), cDNA was in vitro transcribed in aaRNA including amino allyl modified nucleotides (aaUTP). Both dsDNA and aaRNA underwent a purification step using columns provided with the kit. Labelling was performed using NHS ester Cy3 or Cy5 dyes (Amersham Biosciences) that are able to react with the modified RNA. The mRNA quality was checked using RNA 6000 nano chip assays (Agilent Technologies). At least 5 μg of mRNA for each sample was labelled and purified with columns. Equal amounts (0.825 μg) of labelled sample and reference specimens were combined, fragmented and hybridised to oligonucleotide glass arrays representing the Hibiscus rosa-sinensis L. transcriptome. All steps were performed using the In Situ Hybridisation Kit Plus (Agilent Technologies) and followed the 60-mer oligo microarray processing protocol (Agilent Technologies). Then, the slides were washed with the Agilent wash procedure and scanned with a dual-laser Agilent G2505B microarray scanner. A custum gene expression microarray of Hibiscus rosa-sinensis was used. The microarray design format was 4 × 44 K. Each slide contained four microarrays (Supplementary Table S3). The total number of oligos was 45,220 with 1,417 Agilent controls. The length of the probes was 60 bp, and the probes were randomly located on the microarray slide. Five replicates for each probe were used. The labelled RNA was hybridised to the sequences on the chip as described in Janssen et al. (2008). In brief, for each of the two biological replicates, RNA from each treatment was labelled with Cy3 and hybridised in the presence of Cy5-labelled petunia onto the tomato genome set. Both labelling experiments were then repeated so that each RNA population was hybridised four times, twice with Cy5 and twice with Cy3. The data were analysed by fitting linear models to both the log2 Cy5/Cy3 ratios (ratio analysis) and log2 Cy3 and log2 Cy5 intensities (separate channel analysis) using the limma software package (Smyth and Speed 2003).

The design of the chips used is shown in Supplementary Table S1. The microarray results represent an average of three independent biological replicates for each flower organ.

### Validation of genes using quantitative PCR

The microarray transcriptional profiling results were validated using quantitative reverse transcription (RT)-PCR measurements on chosen ethylene biosynthesis genes in different flowers organs (petals and S–S + S) at the different development stages. Primer sequences and quantitative PCR conditions are as reported previously (Trivellini et al. 2011a, b, c). Gene transcript abundance changes showed the same trends as observed in the microarray described above.

### Statistical analysis

The data were subjected to statistical analysis using PRISM 8 software (GraphPad Software, San Diego, CA, USA). Analysis of variance (one-way or two-way ANOVA) was used and means values were separated using the Tukey’s or Bonferroni multiple comparison test (*p* < 0.05).Log2 fold change (FC) > 2 was used as the threshold to determine the significant difference in gene expression. A hierarchical cluster analysis (HCA) was generated in R with hclust and dist methods from stats package, using complete linkage method on euclidean distance matrix on normalized feature values. The heatmaps from ComplexHeatmap of BiocManager were annotated with a word-map expressing the frequency of occurrences of the functional categories in each cluster.

### Supplementary Information

**Additional file 1: Supplementary Table S1**. Effect of different saline irrigation water on *H. rosa-sinensis *(cv. Porto and cv. Sunny wind) plants in term of survival after six weeks.

**Additional file 2. Supplementary Table S2**. List of differential regulated genes among *H. rosa-sinensis* cultivars and floral organs under 100 mM NaCl treatment.

**Additional file 3. Supplementary Table S3**. Design of microarray chips used in this study.

**Additional file 4. Supplementary Table S4**. Microarray validation by qRT-PCR and list of genes used in the qRT-PCR analysis.

**Additional file 5. Supplementary Figure S1**. Agarose electrophoresis gel of total RNA.

## Data Availability

Flowering plant of Hibiscus cultivar Porto and Sunny Wind are commercially available.

## Abbreviations

ABA: Abscisic acid
DEGs: Differential expressed genes
AP2/ERF: APETALA2/Ethylene response factor
ERF-1: Ethylene response factor 1
GH3: Indole-3-acetic acid-amido synthetase
ADH: Alcohol dehydrogenase
NCED: 9-Cis-epoxycarotenoid dioxygenase
qRT-PCR: Quantitative real-time PCR
ROS: Reactive oxygen species
K+: Potassium
Ca2+: Calcium
Na+: Sodium
PI: Performance index
Fv/Fm: Maximum quantum efficiency of photosystem II
