# Supplementary material for: Molecular and physiological responses to salt stress in salinity-sensitive and tolerant Hibiscus rosa-sinensis cultivars
Source: Mol Hortic. 2023 Dec 19;3:28. doi: 10.1186/s43897-023-00075-y (PMC10731769; doi:10.1186/s43897-023-00075-y)
Supplement: Supplementary file 3 — Additional file 3. Supplementary Table S3. Design of microarray chips used in this study. [file 43897_2023_75_MOESM3_ESM.docx]

**Microarray scheme analysis.**

| **Slide 1** |  |  |  |  |  |  |
| --- | --- | --- | --- | --- | --- | --- |
| CTRL OS Y **H3** |  | CTRL PET Y **H5** |  | CTRL OS Y **H3** |  | CTRL OS Y **H3** |
| Vs |  | vs |  | vs |  | DYE SWAP |
| 100mM OS Y **H1** |  | 100mM PET Y **H10** |  | CTRL OS R **H6** |  | CTRL OS R **H6** |
|  |  |  |  |  |  |  |
| **Slide 2 (2nd rep)** |  |  |  |  |  |  |
| CTRL OS Y **H4** |  | CTRL PET Y **H16** |  | CTRL OS Y **H4** |  | CTRL OS Y **H4** |
| vs |  | vs |  | vs |  | DYE SWAP |
| 100mM OS Y **H9** |  | 100mM PET Y **11** |  | CTRL OS R **H7** |  | CTRL OS R **H7** |
|  |  |  |  |  |  |  |
| **Slide 3** |  |  |  |  |  |  |
| CTRL OS R **H6** |  | CTRL PET R **H8** |  | CTRL PET Y **H5** |  | 100mM OS Y **H1** |
| vs |  | vs |  | vs |  | vs |
| 100mM OS R **H12** |  | 100mM PET R **14** |  | CTRL PET R **H8** |  | 100mM OS R **H12** |
|  |  |  |  |  |  |  |
| **Slide 4 (2nd rep)** |  |  |  |  |  |  |
| CTRL OS R **H7** |  | CTRL PET R **H13** |  | CTRL PET Y **H16** |  | 100mM OS Y **H9** |
| vs |  | vs |  | vs |  | vs |
| 100mM OS R **H2** |  | 100mM PET R **H15** |  | CTRL PET R **H13** |  | 100mM OS R **H2** |
|  |  |  |  |  |  |  |
| **Slide 5** |  |  |  |  |  |  |
| 100mM PET Y **11** |  | 100mM PET Y **10** |  | CTRL PET Y **H16** |  | 100mM OS Y **H9** |
| vs |  | vs |  | DYE SWAP |  | DYE SWAP |
| 100mM PET R **14** |  | 100mM PET R **15** |  | CTRL PET R **H13** |  | 100mM OS R **H2** |

**Legend:**

OS= ovary + style-stigma-stamens

PET= petalo

Y= cultivar Sunny wind

R= cultivar Porto

100mM= 100mM NaCl 4 weeks in the treatments

CTRL= control four weeks
