# Supplementary material for: Molecular and physiological responses to salt stress in salinity-sensitive and tolerant Hibiscus rosa-sinensis cultivars
Source: Mol Hortic. 2023 Dec 19;3:28. doi: 10.1186/s43897-023-00075-y (PMC10731769; doi:10.1186/s43897-023-00075-y)

Supplementary Figure 1. RNA integrity assessment using 1% agarose electrophoresis. Electrophoretic profiles of total RNA from *Hibiscus rosa-sinensis* L. floral organs obtained growing the plant under control and saline (100 mM NaCl) conditions. R, ‘Ruby’ cultivar; S w, ‘Sunny wind’ cultivar; S-S-S + O, style-stima and stamen plus ovary.


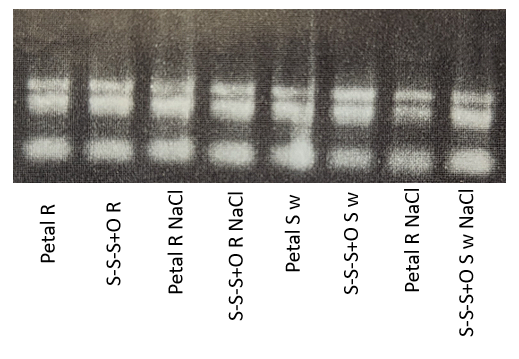

Supplement: Supplementary file 5 — Additional file 5. Supplementary Figure S1. Agarose electrophoresis gel of total RNA. [file 43897_2023_75_MOESM5_ESM.docx]
